# Assessing Consciousness: Of Vigilance and Distractedness

**DOI:** 10.1371/journal.pbio.0030179

**Published:** 2005-04-12

Have you ever walked smack into a parking meter or tripped over something on the sidewalk? Embarrassing as such incidents may be, they're the product of normal brain function. The brain is continuously bombarded with sensory information about the environment but perceives just a fraction of these inputs. The rest—pertinent details or not—is filtered out. It's thought that consciousness emerges from the activity of multiple spontaneous neural processors that run in parallel and connect to a higher order cognitive network that mediates the conscious perception. But this higher order network has limited processing capacity. That means if you're distracted, your brain can't accommodate additional sensory information, like “there's a parking meter in front of you, look out!”

To understand how spontaneous brain processing interacts with higher order cognition, Stanislas Dehaene and Jean-Pierre Changeux modeled the dynamic properties of brain activity with computer simulations. Their simulations show that while spontaneous brain activity sometimes facilitates processing, more often it competes with external stimuli for access to consciousness. Intriguingly, the results of the computer simulations very closely match physiological and psychophysical experimental data and thus shed new light on how intrinsic brain activity modulates conscious perception.

Neurons are simulated in their model as single “integrate and fire” units, integrating the signals received from all connected cells and firing an action potential as soon as their threshold is exceeded. These units are nested in columns, which are multiply linked among themselves and thereby form hierarchical assemblies. Lower columns increase their firing activities upon perception of external stimuli. This excitation propagates upwards to higher processing areas in a bottom-up activation process, but the model also includes, critically, top-down connections that can amplify incoming inputs. Eventually, if the input is strong enough, the reverberating excitation results in “ignition” of the global workspace with all areas simultaneously displaying sustained high firing activities. In the ignited network, the information of the stimulus is globally available; in this simplified model ignition corresponds to the access to consciousness. Most interestingly, the ignition threshold is variable and depends on the intensity of spontaneous activity in the network prior to perception of the stimulus.

In their study, Dehaene and Changeux changed the values of only one input parameter: the ascending neuromodulatory current. This model parameter simulates the effect of various diffuse neuromodulatory systems located in lower regions of the brain, which regulate the transition between awake and asleep states by liberating a diversity of neurotransmitters in the cortex and thalamus, the upper brain regions. The dynamics of the neuronal network could clearly be separated into two broad states of activity. Below a certain threshold for the neuromodulatory current, ignition of the global workspace is not possible, no matter how strong the stimulus—this can be compared to the brain states of comatose patients lacking any sign of consciousness. Above the threshold, however, spontaneous neural activity emerges: firing signals are amplified in feedback loops in single columns of the neuronal network.

With higher vigilance states, weaker external stimuli are able to ignite the global workspace. But paying attention to one thing narrows your perceptive capacity. Once ignited by one stimulus, the network cannot consciously process any others. Dehaene and Changeux propose that spontaneous activity—which operates within an “anatomically distinct set of workplace neurons”—offers an organism a measure of autonomy relative to the external world. While this decoupling of internal thought and external stimuli does have its disadvantages—like that pesky parking meter—it also provides the opportunity for introspection and creativity, which the authors argue is likely to “play a crucial role in the spontaneous generation of novel, flexible behavior.”

